# The Influence of *BRAF* and *KRAS* Mutation Status on the Association between Aspirin Use and Survival after Colon Cancer Diagnosis

**DOI:** 10.1371/journal.pone.0170775

**Published:** 2017-01-26

**Authors:** Martine A. Frouws, Marlies S. Reimers, Marloes Swets, Esther Bastiaannet, Bianca Prinse, Ronald van Eijk, Valery E. P. P. Lemmens, Myrthe P. P. van Herk-Sukel, Tom van Wezel, Peter J. K. Kuppen, Hans Morreau, Cornelis J. H. van de Velde, Gerrit-Jan Liefers

**Affiliations:** 1 Department of Surgery, Leiden University Medical Center, Leiden, The Netherlands; 2 Department of Pathology, Leiden University Medical Center, Leiden, The Netherlands; 3 Comprehensive Cancer Organisation The Netherlands, Eindhoven, The Netherlands; 4 Department of Public Health, Erasmus MC Medical Centre, Rotterdam, The Netherlands; 5 PHARMO Institute for Drug Outcomes Research, Utrecht, The Netherlands; Queen Mary Hospital, HONG KONG

## Abstract

**Background:**

Use of aspirin after diagnosis of colon cancer has been associated with improved survival. Identification of cancer subtypes that respond to aspirin treatment may help develop personalized treatment regimens. The aim of this study was to investigate the influence of *BRAF* and *KRAS* mutation status on the association between aspirin use and overall survival after colon cancer diagnosis.

**Methods:**

A random selection of 599 patients with colon cancer were analyzed, selected from the Eindhoven Cancer Registry, and *BRAF* and *KRAS* mutation status was determined. Data on aspirin use (80 mg) were obtained from the PHARMO Database Network. Parametric survival models with exponential (Poisson) distribution were used.

**Results:**

Aspirin use after colon cancer diagnosis was associated with improved overall survival in wild-type *BRAF* tumors, adjusted rate ratio (RR) of 0.60 (95% CI 0.44–0.83). In contrast, aspirin use in *BRAF* mutated tumors was not associated with an improved survival (RR 1.11, 95% CI 0.57–2.16). *P-value* for interaction was non-significant. *KRAS* mutational status did not differentiate in the association between aspirin use and survival.

**Conclusion:**

Low-dose aspirin use after colon cancer diagnosis was associated with improved survival in *BRAF* wild-type tumors only. However, the large confidence interval of the rate ratio for the use of aspirin in patients with *BRAF* mutation does not rule out a possible benefit. These results preclude *BRAF* and *KRAS* mutation status to be used as a marker for individualized treatment with aspirin, if aspirin becomes regular adjuvant treatment for colon cancer patients in the future.

## Introduction

A significant body of proof has already demonstrated that aspirin has anticancer effects in colorectal cancer (CRC) [1–5]. Randomized controlled trials investigating the cardiovascular benefits of aspirin have shown a significant reduction of CRC risk and mortality [1, 6, 7]. In patients with a history of colorectal adenomas, aspirin has been proven effective in the prevention of these lesions [8]. The most recent meta-analysis of observational studies by Elwood *et al*. found a 25% reduction in colorectal cancer-related deaths and a 20% overall mortality reduction [4]. Altogether, these publications have led to several ongoing randomized controlled trials studying the effect of aspirin on cancer mortality which are currently being conducted globally: the Add-Aspirin trial [9], Adjuvant Aspirin for Colon Cancer (NCT02467582), the ALASCCA trial (NCT02647099), the ASCOLT trial (NCT00565708), and the Aspirin trial (NCT02301286).

If the survival benefits are so obvious, why not prescribe aspirin to all colorectal cancer patients? Because of the side-effects, the use of aspirin is not without risk: common adverse effects are upper gastrointestinal symptoms, and increased bleeding tendency which can cause epistaxis, gastrointestinal bleeding or purpura [5]. Low-dose aspirin, indicated for secondary cardiovascular risk management, roughly doubles the incidence of gastric bleeding. One or two patients in every thousand are likely to have a gastric bleed each year. The bleeding risk increases with age and in patients 80 years and older, this may even be seven per 1000 people per year [10]. Identifying which patients may benefit from aspirin treatment may help develop effective personalized treatment regimens, thereby reducing overtreatment and negative side effects associated with aspirin. Several biomarkers have been suggested to be differentiating in the association between aspirin and improved cancer survival, however results are very heterogeneous [5]. Despite promising data, the clinical use of any biomarker in general practice is lacking, and currently only *KRAS*, *BRAF* and microsatellite instability are currently used in the diagnosis and treatment of colorectal cancer [11].

Mutated *BRAF* and *KRAS* oncogenes, both members of the Mitogen Activated Protein Kinase (MAPK) pathway, are respectively observed in approximately 10–20% and 35–42% of the sporadic colorectal cancers [11–13]. Mutated *BRAF* and *KRAS* have been shown to influence MAPK signaling, resulting in upregulation of Prostaglandin-endoperoxide synthase 2 (PTGS2, also known as COX-2) [14]. *BRAF* mutations are associated with the presence of high microsatellite instability, the molecular hallmark of Lynch syndrome [15]. Evidence from the CAPP2 trial demonstrated that individuals with Lynch syndrome could be recommended to consider taking daily low-dose aspirin [16]. With this link and the known crosstalk between the phosphatidylinositol-4,5-bisphosphate 3-kinase catalytic subunit alpha (PIK3CA) pathway and MAPK pathway, the assessment of *BRAF* and *KRAS* mutational status as molecular biomarker for the survival benefit associated with the use of aspirin could be a next step to unravel the biological effect of aspirin in colon cancer [17].

Therefore, the aim of this study was to investigate the association of low-dose aspirin use after colon cancer diagnosis and survival of patients according to *BRAF* and *KRAS* mutation status.

## Materials and Methods

### Study cohort

Data on low dose aspirin use (80–100 mg), derived from the PHARMO Database Network (PHARMO, Netherlands), were linked to the Eindhoven Cancer Registry (ECR). The validity of the linkage of these cohorts was described previously [18]. The ECR serves about 1.5 million inhabitants in the southern region of the Netherlands and is part of the nationwide Comprehensive Cancer Organisation (IKNL). The PHARMO Database Network is a population-based network and combines data from different healthcare settings in the Netherlands. The Outpatient Pharmacy Database was used for this study, which comprises drug dispensing records from all community pharmacies. The records in this database contain information on the type of product, date prescribed, dose and regimen, quantity, and route of administration. Drugs are coded using the Anatomical Therapeutic Chemical classification [19]. The Comprehensive Cancer Organisation is obliged to work according to the law on data protection; informed consent of the patients for this specific study was not applicable.

As previously published, aspirin initiated or continued after diagnosis was associated with improved survival for patients with colon cancer, but not for patients with rectal cancer, in our cohort [20]. Therefore, only patients with colon cancer were included in this study.

The vital status of patients (alive/dead) was established from medical records or through linkage of cancer registry data with the municipal population registries. As information on hospital dispensing was not available, follow-up started 14 days after diagnosis of colorectal cancer (T0), and was continued until last contact date (January 2012), date of loss to follow-up, or date of death—whichever occurred first.

**Fig 1:**
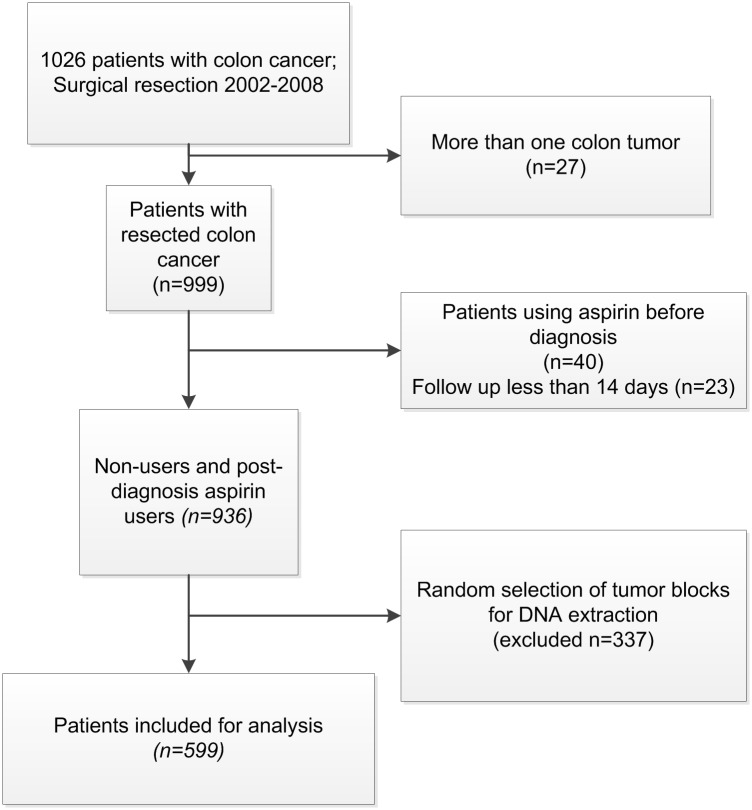
Flowchart of patients selected for analysis.

Patients who only used aspirin before diagnosis were also excluded (n = 40, see Fig 1). Non-users were classified as those who never had a dispensing for aspirin or had a dispensing for less than 14 days after diagnosis of colon cancer. Users were defined as those who had been given a dispensing of aspirin for 14 days or more after a colon cancer diagnosis. The median duration of one dispensing was 30 days and the mean dispensing number was 12 (range 1–220).

### BRAF and KRAS tumor mutation analyses

The ECR-PHARMO cohort, as previously published by Bastiaannet *et al*, contained 3,586 patients [20]. Of this cohort, Formalin-Fixed Paraffin-Embedded (FFPE) tumor tissues were retrieved of 1,026 colon cancer patients who underwent a surgical resection between 2002 and 2008 [21]. Twenty-seven patients with more than one colon tumor at the time of diagnosis were excluded from this cohort (Fig 1). Additionally, 63 patients were excluded because they used aspirin before diagnosis or with a follow up less than 14 days. Of these patients, 599 patients were randomly selected with a ratio 1:2 for aspirin user: non-user, as was previously described [22].

No significant demographic differences were calculated between the total cohort (n = 999) and the randomly selected patients (n = 599) [22].

Of the included patients (n = 599), tumor areas on hematoxylin and eosin (H&E) stained tumor sections were marked by an experienced pathologist/researcher. Guided by the H&E-stained slides, 1–2 punches with a diameter of 2.0 mm diameter and variable length were taken from the tumor focus, followed by DNA extraction as described by de Jong *et al* [21]. For determination of *KRAS* and *BRAF* mutations status, hydrolysis probes assays were performed for the major known mutations (hotspots) in codon 600 for *BRAF*, c.1799T>A; p.V600E and codon 12 and 13 for *KRAS*; c.34G>A; p.G12S, c.34G>C; p.G12R, c.34G>T; p.G12C, c.35G>A; p.G12D, c.35G>C; p.G12A, c.35G>T; p.G12V, c.38G>A; p.G13D and c.37G>T; p.G13C, as previously described [21]. Hydrolysis probe assays were analyzed using qPCR analysis software (CFX manager version 3/0, Bio-Rad). Mutation detection was performed by two independent observers (M.R. and R.E.). All primers used for the assays were previously described [23].

### Statistics

Statistical analyses were performed using the statistical packages SPSS (version 20.0 for Windows, IBM SPSS statistics) and Stata (version 12 for windows, StataCorp LP). Statistical tests were two-sided and considered significant at a *p*-value below 0.05.

A parametric survival model with an exponential (Poisson) distribution was used, with the use of aspirin as time varying covariate. This method prevents the introduction of time-related biases [24]. Non-users were defined from T0 until date of death or end of follow-up. Patients were considered aspirin users from the moment of first prescription, mimicking an intention-to-treat analysis. In order to investigate differential associations of aspirin use with overall survival by tumor molecular subtype, stratified analyses were performed for *BRAF* wild-type / *BRAF* mutation and *KRAS* wild-type / *KRAS* mutation, followed by an interaction analysis. The interaction analysis was performed by including a cross product of *BRAF* mutation status in the survival analysis and the use of aspirin and significance was assessed with the Wald test.

Adjustments for potential confounders were made for sex, age (groups), stage (pathological stage and clinical stage if pathological stage was unknown), adjuvant chemotherapy (yes/no), co-morbidity (yes/no) and tumor grade.

Survival curves were calculated according to the Simon-Makuch method, an alternative for Kaplan Meier, with the ability to include time-varying covariates [25].

A subgroup analysis was performed by excluding patients with stage IV disease.

## Results

Fig 1 shows the flowchart of the study population eligible for analysis. In this cohort, 29.9% (179/599) of the patients were defined as aspirin users. Of the 179 patients who used aspirin after diagnosis, 27 patients started using aspirin after diagnosis and 155 used already aspirin at diagnosis. In total, 267 deaths were recorded before January 2012.

DNA was extracted from FFPE tumor tissues and *BRAF* mutation status (wild-type/mutation) was successfully established in 98% of the samples. A *BRAF* mutation was found in 17% (102/599) and a *KRAS* mutation was observed in 35% of colon tumors (212/599), in accordance with previous studies [11–13, 26, 27].

Table 1 summarizes the clinical characteristics of the patients included in the analysis. Both age and frequency of comorbidities were found to be higher in the group of aspirin users compared to non-users. Lower disease stage and male sex were more often observed in aspirin users compared to non-users.

**Table 1:** Baseline Characteristics of the cohort.

		All patients		Non-users		Aspirin users	
		n	%	n	%	n	%
	Total	599	100	420	100	179	100
**Sex**	Male	327	54.6	215	51.2	112	62.6
	Female	272	45.4	205	48.8	67	37.4
**Age category**	<65	189	31.6	158	37.6	31	17.3
	66–74	189	31.6	118	28.1	71	39.7
	75 and older	221	36.9	144	34.3	77	43.0
**Year of diagnose**	2002–2004	300	50.1	208	49.5	92	51.4
	2005–2007	299	49.9	212	50.5	87	48.6
**Disease stage**	I	95	15.9	57	13.6	38	21.2
	II	237	39.7	166	39.7	71	39.7
	III	176	29.5	121	28.9	55	30.7
	IV	89	14.9	74	17.7	15	8.4
**Comorbidity**	No	209	34.9	176	41.9	33	18.4
	Yes	342	57.1	202	48.1	140	78.2
	Missing	48	8	42	10	6	3.4
**BRAF mutation analysis**	Wild-type	497	83	347	82.6	150	83.8
	Mutation	102	17	73	17.4	29	16.2
**KRAS mutation analysis**	Wild-type	387	64.6	274	65.2	113	63.1
	Mutation	212	35.4	146	34.8	66	36.9

More detailed patient characteristics according to aspirin use and *BRAF* and *KRAS* mutation status are shown in S1 Table. Aspirin use was equally distributed: 29% in patients with wild-type *BRAF* tumors, 27% in patients with mutated *BRAF* tumors, and 29% in patients with *KRAS* wild-type and 31% in patients with mutated *KRAS* tumors.

**Fig 2:**
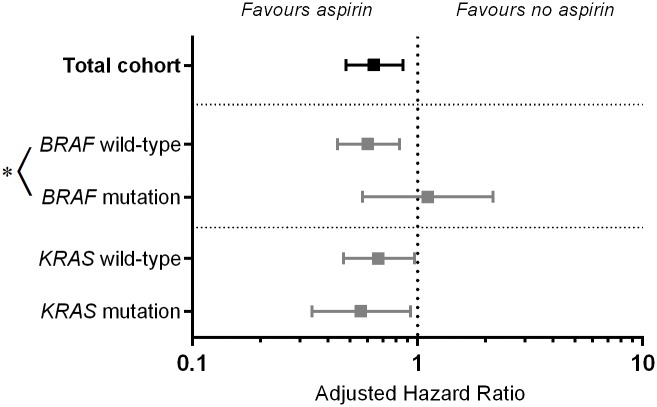
Overall survival analysis for patients using aspirin versus patients not using aspirin, grouped according to mutation status. * Test for interaction not significant.

**Fig 3:**
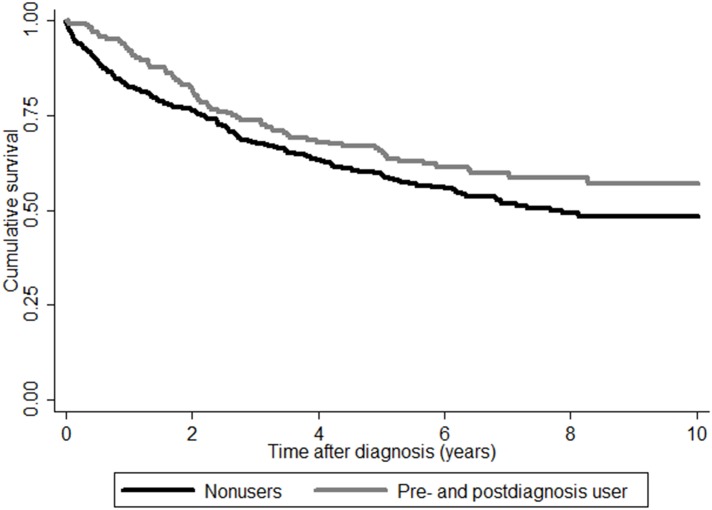
Survival curves for aspirin users versus non-users according to the Simon-Makuch method.

As shown in Table 2 and Fig 2 aspirin use after diagnosis was associated with an improved overall survival in the total cohort (n = 599) (crude Rate Ratio (RR) 0.73, 95% Confidence Interval (CI) 0.56–0.97, adjusted RR 0.64 (95% CI 0.48–0.86)). Fig 3 shows the survival curves for these patients.

**Table 2:** Rate Ratio for Death (Time-Dependent Analysis Overall Survival), According to Tumor *BRAF* and *KRAS* mutation status, and use or no use of Aspirin after Diagnosis.

		n	Events	Univariate		Multivariate	
				RR (95%CI)	P-value	RR_a_ (95%CI)	P-value
**Overall**		**599**					
	No aspirin use	420	199	*1*.*00 (reference)*	**0.03**	*1*.*00 (reference)*	**0.003**
	Aspirin use	179	68	**0.73 (0.56–0.97)**		**0.64 (0.48–0.86)**	
**BRAF mutation status**							
Wild-type		*497*					
	No aspirin use	347	159	*1*.*00 (reference)*	0.05	*1*.*00 (reference)*	**0.002**
	Aspirin use	150	55	0.74 (0.54–1.00) _b_		**0.60 (0.44–0.83)**	
Mutation		*102*					
	No aspirin use	73	40	*1*.*00 (reference)*	0.34	*1*.*00 (reference)*	0.77
	Aspirin use	29	13	0.74 (0.39–1.38) _b_		1.11 (0.57–2.16)	
**KRAS mutation status**							
Wild-type		*387*					
	No aspirin use	274	130	*1*.*00 (reference)*	0.11	*1*.*00 (reference)*	**0.03**
	Aspirin use	113	43	0.75 (0.53–1.06)		**0.67 (0.47–0.97)**	
Mutation		*212*					
	No aspirin use	146	69	*1*.*00 (reference)*	0.14	*1*.*00 (reference)*	**0.03**
	Aspirin use	66	25	0.71 (0.45–1.11)		**0.56 (0.34–0.93)**	

Significant values are printed in bold

^a^ Adjusted for age, comorbidity, grade, stage and chemotherapy

^b^ P-value for interaction = 0.99

For patients with a *BRAF* wild-type tumor, aspirin use after diagnosis showed a RR for overall survival of 0.74 (95% CI 0.54–1.00), and when adjusted for potential confounders this effect was more pronounced with an adjusted RR of 0.60 (95% CI 0.44–0.83, *p* = 0.002, Fig 3). For patients with *BRAF* mutated tumors, aspirin use after diagnosis was not associated with an improved survival (adjusted RR 0.74, 95% CI 0.39–1.38, *p* = 0.34). The Wald test showed a *P* for interaction of 0.99, which suggests that the difference found between the group of patients with a *BRAF* wild-type or mutation is based on chance.

For patients with a *KRAS* mutated tumor and patients with a *KRAS* wild-type tumor, aspirin use after diagnosis was associated with an improved overall survival in the multivariate analysis (*KRAS* wild-type RR 0.68 (0.67 95%CI 0.47–0.97) and *KRAS* mutant RR 0.56 (95% CI 0.34–0.93)), (Table 2, Fig 3).

The results from the subgroup analysis that excluded patients with stage IV can be found in S2 Table.

## Discussion

### Overview of findings

Increasing attention is paid to a personalized treatment approach, by stratifying patients into subgroups based on biomarkers. This study investigated whether the survival benefit observed in patients with colon cancer using aspirin could be associated with *BRAF* or *KRAS* mutational status. This study found that *BRAF* mutation status and *KRAS* mutation status were not distinctive in the association between low-dose aspirin use and a survival benefit in patients with colon cancer. In the multivariate analysis, patients with wild-type *BRAF* tumors, aspirin use after diagnosis was associated with a significantly better outcome. However, the crude hazard ratios in both groups (*BRAF* wild-type and mutation) are equal and the *P*-value for interaction was non-significant. Because no statistical interaction was observed, the distinctiveness of *BRAF* mutational status on the association between aspirin use and survival in the multivariate analysis could very well be based on chance. Therefore, it could not be concluded from this study that patients with *BRAF* mutated tumors should be withheld from using aspirin. The subgroup analysis in patients with stage I-III colon cancer showed a reduced effect size. However, due to limitations in power, no firm conclusions can be drawn from the results of this subgroup analysis.

### Comparison with other studies

Nishihara *et al* [26] previously studied the effect of *BRAF* mutational status on colorectal cancer incidence and survival in patients using aspirin. *BRAF* mutational status showed to be of influence on the incidence of colorectal cancer. *BRAF* wild-type was associated with a lower risk of colorectal cancer, multivariable hazard ratio; 0.73 (95% CI 0.64–0.83) whereas *BRAF* mutated tumors did not show a reduced risk of colorectal cancer (HR 1.03, 95% CI 0.76–1.38). A survival analysis in this study was performed as an exploratory analysis, and in both subgroups (*BRAF* mutation and wild-type tumors) no association between the use of aspirin and improved survival was found, in line with our study.

### Strengths and limitations

Our study has several strengths. To the best of our knowledge, no studies have assessed the association between *KRAS*, aspirin and survival in patients with (colon) cancer. Information regarding aspirin use and dose was derived from prescriptions rather than patient recall, resulting in a precise definition of regular aspirin use. By using a time-varying covariate for the use of aspirin, the risk of non-differential misclassification is reduced [24]. Lastly, a robust and reliable method was used to determine *BRAF* and *KRAS* mutational status, resulting in a 98% successful determination of mutational status and therefore a relatively large cohort.

However, several limitations must be acknowledged. First, over-the-counter aspirin use and adherence was unknown and could be a potential source of bias. Nevertheless, it has been shown that pharmacy data can give valid estimates, despite over-the-counter availability of aspirin [28]. It seems unlikely that a large fraction of patients bought aspirin over-the-counter: low-dose aspirin is only indicated for secondary cardiovascular prevention in the Netherlands and therefore this should always be made available through a doctor’s prescription. The main reason for over-the-counter purchase of aspirin is its use as analgesic, however low-dose aspirin does not suffice as analgesic. Moreover, the possible benefits of aspirin as treatment for cancer were not widely known during the analysis period.

Second, this is a retrospective study in which patients were not randomized. Even after adjustment for potential confounders, residual confounding may still be present. Confounding by indication could, in general, have resulted in overestimation of the results. For cancer patients to be prescribed aspirin, patients should have a cancer prognosis which outweighs the risk of cardiovascular disease. Patients to whom this does not apply should, in theory, not be prescribed aspirin. These patients are then assigned into the non-user group which could have resulted in an overestimation of the association between aspirin use and survival. The variation in length of use of aspirin and the moment patients start using aspirin makes it difficult to conclude any causality from this study, only associations were observed. Therefore, the current ongoing randomised controlled trials are highly warranted. However, this is a limitation of all retrospective studies.

Third, no information regarding disease-specific survival was available in this study. However, a large meta-analysis of individual patient data found that the benefit of patients using aspirin as secondary prevention for cardiovascular disease is only 0.91 (95% CI 0.82–1.00) [29]. This can therefore not fully explain the observed overall survival benefit for the aspirin users in the current study.

To the best of our knowledge, this study is one of the largest cohorts analyzing the association between the use of aspirin, overall survival in colon cancer patients and mutational status of *BRAF* and *KRAS*, however numbers were too small for any additional subgroup analyses.

### Clinical implications

Precision medicine has gained more attention over the last couple of years and multiple publications were dedicated to the discovery and development of clinical prognostic and predictive biomarkers [11]. Nevertheless, conflicting results have been observed for every previous appointed biomarker regarding the association between aspirin use and survival. Proposed biomarkers associated with aspirin use and survival are COX-2, HLA class I, PIK3CA mutation status and several specific genetic profiles [22, 30–32]. Mutations in *BRAF* and *KRAS*, acting in the *RAS-RAF-MAPK* kinase cascade and mutated PIK3CA, acting in *PI3K-PTEN-AKT* signaling pathway, are known for their contribution to the development of CRC and are associated with cancer prognosis [11, 33]. The strong survival benefit in patients with a PIK3CA mutation can only partly explain the effect of aspirin found in the general cancer population. The magnitude of the clinical benefit as found in CRC cohorts, cannot be explained by patients with a PIK3CA mutation solely, because of the low mutation frequency (15%). Therefore, additional biological processes must be responsible for the effect of aspirin on survival.

In this study we were focusing on the *RAS-RAF-MAPK* cascade, known for the crosstalk with the PIK3CA pathway, in relation to aspirin use [17]. No differentiating effect of aspirin use in *BRAF* or *KRAS* mutated tumors could be detected. It could be (cautiously) concluded that biomarkers from the *RAS-RAF-MAPK* cascade and an activated PI3K-PTEN-AKT signaling pathway may not be able to fully unravel the complexity and versatility of the aspirin effect on cancer. Therefore, the evidence points more towards a generalized, systemic effect [5, 34].

One suggested hypothesis is the role of aspirin as thrombocyte aggregation inhibitor [34]. By inhibiting the aggregation of thrombocytes, which naturally shape around circulating tumor cells, the immune system is able to detect and subsequently clear tumor cells from the circulation. Another hypothesis could be found in the anti-inflammatory effects of aspirin [35]. In the past years, several publications focused on the identification of subtypes of colorectal cancer, highlighting the heterogeneity of the disease and aiming to improve optimal allocation of treatment modalities [11, 36, 37]. Linnekamp *et al* advocate that the development of new agents should take place in a disease sub-type-specific fashion and in that manner generate more effective therapies [36]. These subtypes could also be the key to personalized treatment with aspirin. With this information and growing consensus on these subtypes, new research could focus on the effect of aspirin in (inflammatory) specific subtypes.

## Supporting Information

S1 Table Baseline Characteristics of the Colon Cancer Patients according to *BRAF* and *KRAS* mutation status and aspirin use. _a_ p-value for aspirin users vs nonusers.
(XLSX)

S2 Table Rate Ratio for Death (Time-Dependent Analysis Overall Survival), According to Tumor BRAF and KRAS mutation status, and use or no use of Aspirin after Diagnosis, Patients with stage IV disease excluded. Significant values are printed in bold. _a_ Adjusted for age, comorbidity, grade, stage and chemotherapy.
(XLSX)
